# Graft-Site Morbidity in Anterior Cruciate Ligament (ACL) Reconstruction: A Scoping Review of Assessment Techniques and Outcomes

**DOI:** 10.7759/cureus.103565

**Published:** 2026-02-13

**Authors:** Peter M Ciari, Jillian Day, Serena Chen, Karen Bontekoe, Edward Merino, Joshua Karron

**Affiliations:** 1 Research, California Health Sciences University - College of Osteopathic Medicine, Clovis, USA; 2 Biomedical Education, California Health Sciences University - College of Osteopathic Medicine, Clovis, USA

**Keywords:** anterior cruciate ligament (acl), anterior cruciate ligament (acl) reconstruction, bone-patellar tendon-bone grafts, donor site morbidity, quadriceps tendon graft, systematic scoping review

## Abstract

Graft-site morbidity is a critical yet often overlooked aspect of anterior cruciate ligament reconstruction (ACLR). While autografts such as bone-patellar tendon-bone (BPTB) and quadriceps tendon (QT) have been shown to provide effective mechanical stability, the functional and quality-of-life outcomes associated with graft harvesting location warrant greater attention. This scoping review evaluates the existing methodologies for assessing graft-site morbidity, focusing on anterior knee pain, paresthesia, and muscle deficits. Articles were screened from January 1, 2000, to June 12, 2024, returning 609 unique references to graft-site morbidity. A systematic search identified 43 studies meeting inclusion criteria, revealing significant variability in assessment methods and reporting standards. This review underscores the need for a uniformly administered series of techniques to measure graft-site morbidity, enabling meaningful comparisons and improving patient-centric surgical decision-making. Future investigations should prioritize standardizing assessment tools to assess graft selection and optimize holistic patient outcomes.

## Introduction and background

Significant research exists on the functionality of the anterior cruciate ligament (ACL) following anterior cruciate ligament reconstruction (ALCR), and currently graft site is determined by physician preference. This scoping review focuses on the importance of graft-site morbidity, which leads to poor functional outcomes like anterior knee pain, paresthesia, and muscle deficits. 

From the reviewer's initial search into the topic, three types of ACLR predominate the field today: semitendinosus (ST), bone-patellar tendon-bone (BPTB), and quadriceps tendon (QT) [1]. The literature supports the integrity of all three graft options as ACL analogs, suggesting that the evaluation of patient outcomes should follow other considerations. Graft-site morbidity is used interchangeably with donor-site morbidity and, for the purpose of this review, will be defined as adverse outcomes arising from the graft harvesting that may or may not result in functional deficits that persist at one year since surgery. When surveyed, nearly half of the surgeons who responded considered graft-site morbidity as "very or fairly important" [2]. This aspect of patient outcomes should not be disregarded, and its influence on patient recovery could help surgeons decide between techniques.

The focus on the surgical outcomes of the graft is paramount; however, graft-site morbidity directly impacts the functional biomechanics of the surrounding harvested tissue causing frequent negative associations. A systematic review of graft-site morbidity does not currently exist, limiting the determination of which technique leads to overall better morbidity outcomes. The selection of a graft site should complement the nuance of each patient's lifestyle and goals. Some negative outcomes of ACLR that would impede a patient's lifestyle include anterior knee pain, paresthesia, and muscle weakness [3-5]. These metrics were used as the basis for this systematic review. In total, 43 peer-reviewed manuscripts that met the inclusion criteria were identified. Of those manuscripts, very few considered more than one or two aspects of graft-site morbidity. The current inconsistencies in study design prevented extraction and consolidation of data, reflecting the need for a standard set of protocols. The authors, therefore, will review the current techniques for assessing the outcomes of each graft site to provide a comprehensive understanding of proper selection criteria.

Currently, the authors have identified six current systems related in some part to graft-site morbidity in the International Knee Documentation Committee (IKDC), Cincinnati, Shelbourne-Trumper, Noyes, and Lysholm scales. Of these scoring systems, only one provides a comprehensive framework to accurately measure graft-site morbidity. The Shelborne-Trumper scale assesses sporting or daily living activities, prolonged sitting, stair climbing, and kneeling [6]. The above factors contribute significantly to a patient's overall satisfaction with ACLR recovery. With this information, the following essential components of graft-site morbidity can be identified: anterior knee pain, paresthesia, and muscle strength.

In this scoping review, 43 manuscripts have been identified that specifically address the above criteria and outcomes related to graft-site morbidity. This review focuses only on papers related to QT or BPTB. Included studies had a minimum of one year of follow-up, involved male and female participants with an average age of 30±10 years, and were published between 2000 and 2024. This review first uses these criteria to describe anterior knee pain, kneeling ability, paresthesia, functional hop test, and range of motion. It then explores tools to investigate muscle strength including electromyography and dynamometer. This review concludes with a discussion of incorporating these criteria into scoring systems.

## Review

Methods

**Figure 1:**
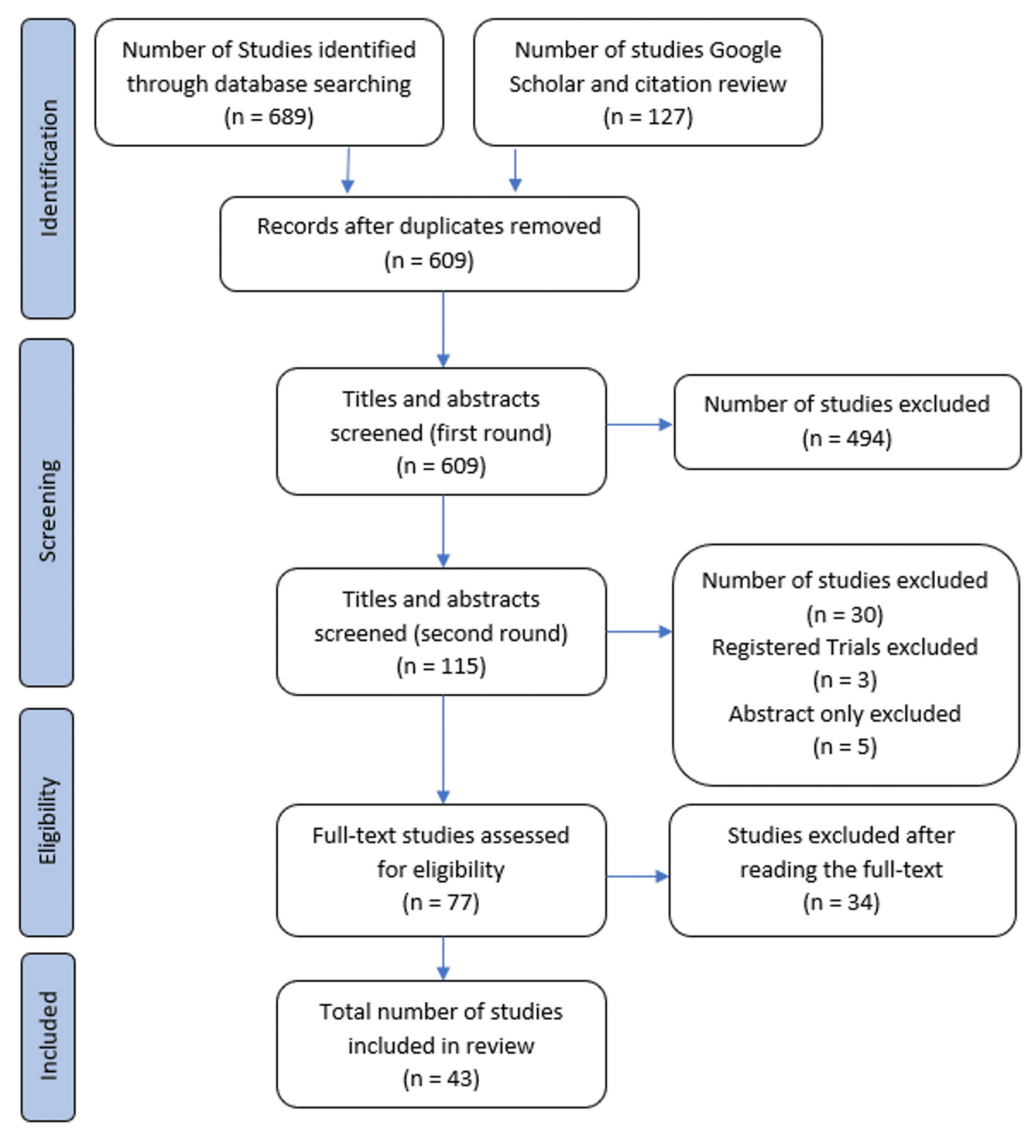
PRISMA chart depicting search protocol. PRISMA: Preferred Reporting Items for Systematic Reviews and Meta-Analyses.

In accordance with the Preferred Reporting Items for Systematic Reviews and Meta-Analyses (PRISMA) Extension for Scoping Review guidelines (PRISMA-ScR) [7], the authors consulted a health sciences librarian to complete a systematic search of PubMed, Embase, the Cochrane Library, and Google Scholar from January 1, 2000, to June 12, 2024. The computer-based literature searches used these keywords: "anterior cruciate ligament AND (donor site morbidity OR harvest site morbidity OR graft site morbidity)." The search was complemented by manually screening reference lists of retrieved articles and relevant reviews to identify additional potentially eligible studies missed by the search strategy. This comprehensive search resulted in 609 potential articles and registered trials (Figure 1). 

Studies included were those with male and female participants with an average age of 30±10 years. A study required at least 20 participants per group without significant comorbidities. Only autografts were considered, specifically BPTB or QT. ST could be included if used in conjunction with one of the aforementioned grafts but not independently. The study's follow-up duration needed to be at least one-year post-surgery. Participants must have recovered extensor function. Studies needed to quantify graft-site morbidity using measures such as the kneeling test, hypoesthesia (area of numbness), and pain ratings. Studies using binary measures (e.g., "yes" or "present") may still be included but noted as examples where standardized scales would be beneficial. Studies from any country were eligible as long as they involved the specified autografts and were accessible in English. Studies primarily discussing revision were excluded, but studies that included revisions as an outcome were not excluded. Studies were excluded if there were concurrent injuries to other knee ligaments. Only primary research articles were included; meta-analyses, reviews, commentaries, or letters were excluded to avoid duplicating data points.

Initial screening by title and abstract was conducted independently by author KB. Following this, authors JK, PC, JD, and SC reviewed the remaining abstracts and trial registries. It was determined that trial registries were not useful for this review. The librarian retrieved the remaining 77 full-text articles for review against the inclusion/exclusion criteria. Any discrepancies in selection were resolved by consensus among the authors, resulting in a final manuscript count of 43. Consistent with PRISMA-ScR guidance, a formal risk of bias assessment was not performed, as the primary objective of this scoping review was to map the extent, range, and methodological heterogeneity of graft-site morbidity assessment rather than to evaluate effectiveness.

Results

*Anterior Knee Pain* 

**Figure 2:**
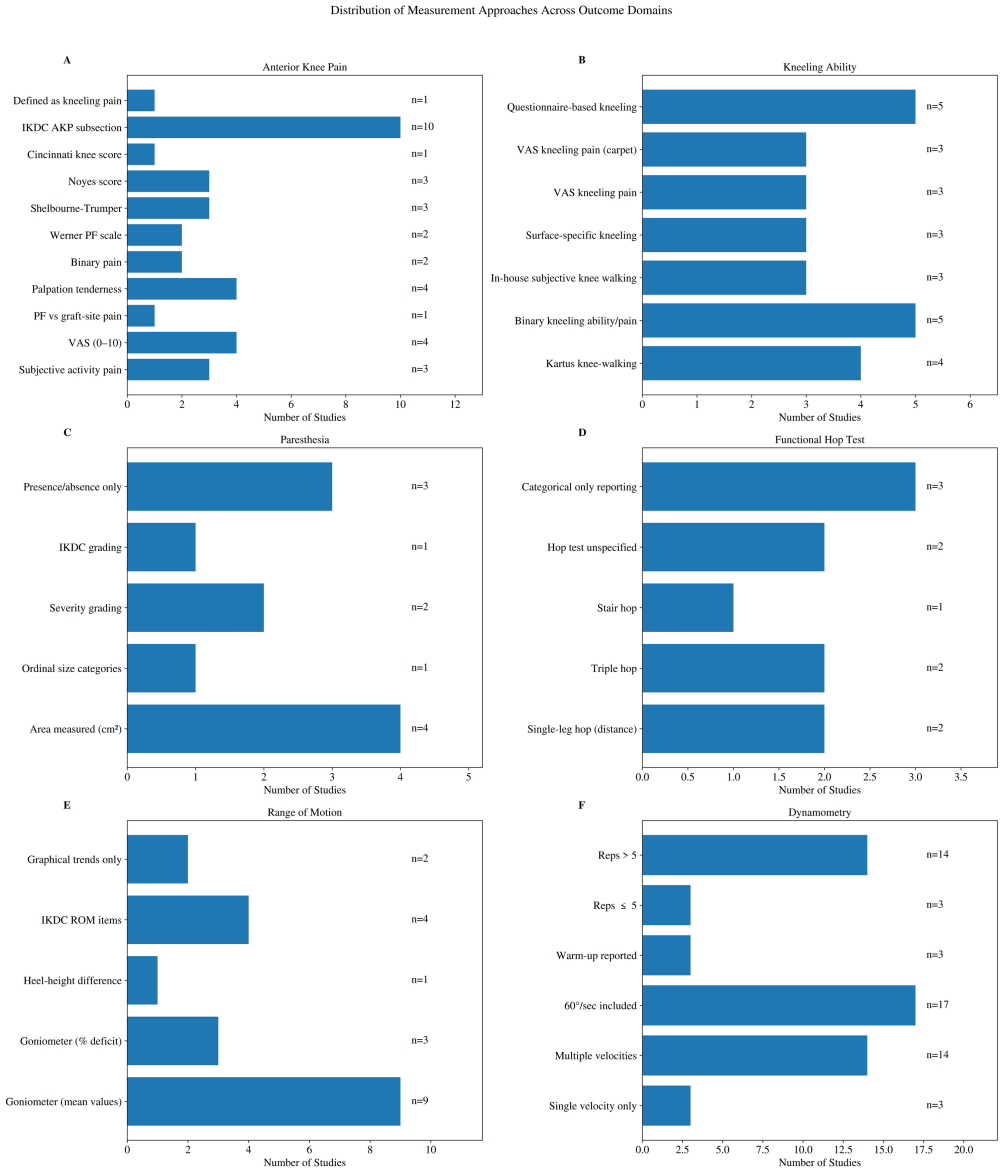
Summary of the distribution of measurement approaches across outcome domains. IKDC: International Knee Documentation Committee; AKP: anterior knee pain; ROM: range of motion; VAS: visual analog score; PF: patellofemoral.

Across included studies, 30 studies reported anterior knee pain outcomes. Definitions and measurement approaches varied across studies (Figure 2A). Three studies reported subjective knee pain when patients complained of pain during "stair-walking, sitting with the knees in 90° of flexion, and during or after activity" [8-10]. Four studies used a visual analog scale for pain and rated from 0 to 10 [11-14], but did not assess pain during activity. A single study differentiated between "patellofemoral syndrome-type pain" and "pain in the graft harvest site (quadriceps tendon tendinopathy)" [15]. Tenderness of skin or graft site on palpation was a frequent measure, though its description varied [16-19]. Binary assessment of pain was measured in two manuscripts specifying if the pain was intermittent [20], or "pain related functional deficiency" [21]. Anterior knee pain incorporated into a composite score in the overarching questionnaires limited analysis. Two studies used the patellofemoral pain scale attributed to Werner [22,23]. Three used the Shelbourne-Trumper questionnaire [24-26]. Three manuscripts used Noyes [26-28], and one used the Cincinnati knee score [29]. Ten total studies used the IKDC anterior knee subsection specifically in analyzing the results of the autograft [19,24,29-36]. Many other studies used the IKDC overall score or other comprehensive questionnaires, but they were only considered relevant if the study used it specifically for anterior knee pain or one of the other categories the reviewers were exploring. Finally, while kneeling tests are their own category for this review's considerations, one paper considered anterior knee pain to be the presence of kneeling pain and knee walking pair [37]. After anterior knee pain, the ability to walk on the knees or comfortably kneel was the next outcome to consider and is reviewed in the subsequent section. 

*Kneeling Ability* 

A literature review of kneeling ability found 23 unique manuscripts that assessed the presence of kneeling pain after autograft to determine graft-site morbidity (Figure 2B). These tests had less variation than with anterior knee pain, but many were scored differently. Four studies scored knee walking as described by Kartus [8,15,23,38]. Five more studies assessed the binary metric of ability to kneel or pain with kneeling [17,18,29,37,39]. Three studies used their own in-house qualitative subjective scoring for the ability to do knee walking [9,10,40]. Three studies differentiated the ability to kneel on different surfaces with varying hardness [16-18]. Three studies used a visual analog scale to describe pain when kneeling [11,12,41]. Three other studies specified "kneeling pain on a standard carpet surface" and utilized a visual analogue scale [19,33,35]. Five studies used other questionnaires to assess kneeling ability, such as Noyes score [27,28], Shelbourne-Trumper [26], and IKDC [34,36]. While kneeling ability tests had less variation than general assessments of anterior knee pain, the reviewers found discrepancies in reporting or measuring strategy. Pain is not the only metric for graft-site morbidity; loss or change in sensation is a serious iatrogenic complication.

*Paresthesia* 

Regarding variation in the measuring of paresthesia, 11 studies considered change in sensation (Figure 2C). However, the methods used to determine what kind of change in sensation, the amount of skin affected, and the degree of change in sensation varied significantly. Four studies measured the square centimeters of the affected area [8,10,22,25], but even among them, there were deviations. Of these, one study had a physiotherapist administer a survey that included a drawing of a knee that the practitioner was to mark [10], in square centimeters, the area of "sensory disturbance "light touch and pinprick." Another study had patients mark the areas of sensory disturbance on their leg with a dermographic pen and then use the major axes to calculate square centimeters [22]. The last two studies that considered quantitative area instructed a provider to palpate the knee of the patient and then calculate square centimeters [8,25]. Of the other studies measuring paresthesia, only one other considered the area, but it was performed by surveying the patient and choosing between "none, between a dime and a quarter, palm of hand, or too big to measure" and then assigning each answer choice a numerical points value [16]. The other studies that considered paresthesia did not consider the size of the change. One study asked the patient to choose between "no sensory loss, disturbed sensitivity, and lost sensitivity", which was then assigned a numerical value [23]. Another paper asked patients to rate their paresthesia as "numbness of skin (none, slight, moderate, severe)" [17]. One study included numbness with tenderness and irritation as scored by the IKDC grading [33]. The final three studies that investigated paresthesia considered the presence or absence of skin numbness [18], but one of them included it as a yes or no question on a survey and the other two simply stated that no numbness was self-reported by patients [13,19]. Thus, there are two metrics used to measure paresthesia, which are the existence versus the size of paresthesia present at the graft site. An important aspect of iatrogenic injury remains in the loss of muscle function, as measured in the next sections.

*Functional Hop Test* 

One easy and cost-efficient way to measure function in relation to graft-site morbidity is the hop test, of which there were seven references to a functional hop test (Figure 2D). The single-legged hop test for distance evaluates functional strength, an aspect of graft-site morbidity, whereas a hop test that measures side-to-side knee shift measures laxity of the ACL itself. This results in numerous inconsistencies across the studies, as some do not fully describe the hop test they utilized [20,27], and others did not present quantitative results of their tests, instead simply describe the frequency of normal findings and state the lack of significant differences [27,33,34]. There was also a slight variation in the hop tests themselves, as some studies chose to add the triple hop and stair hop to assess function [12,28]. Only two studies provided raw data of the linear distance hopped in centimeters [28,42]. While the functional hop test appears simple to complete, the mixed presentation of results between studies limits its utilization as a greater data set. Range of motion is one of the essential pieces of physical examination and even more necessary when evaluating graft-site morbidity.

*Range of Motion* 

Harvesting of graft tissue can incur the formation of scar tissue that limits range of motion, which can be readily measured in any physical exam. Eighteen studies assessed range of motion in the context of ACLR (Figure 2E). Most studies utilized the goniometer measured to the nearest five degrees to evaluate differences in extension and flexion, but four manuscripts did not mention their methods of evaluating range of motion deficits [15,18,30,41]. One study evaluated extensor deficits by measuring prone heel height differences [11]. There was even more variation in the presentation of the results, as five studies simply described the average extension and flexion differences and the lack of statistical significance in the range of motion deficits [8,14,15,28,41]. In contrast, three other manuscripts specifically charted the percentage of flexion and extension deficit [11,23,37]. Two additional studies charted the number of patients with flexion and extension deficits [9,19]. Four more studies pulled the range of motion results from the IKDC survey and described the percentage of patients with lack of flexion or extension [17,27,34,40]. In an additional study with two follow-up manuscripts, the authors graphed the progression of the extension deficit over the years, allowing for visualization of change and improvement but lacked individual data points to be used in comparison to other studies [33,35]. While the measurement of range of motion has consistent methods, the presentation of the data limited the interpretation of graft site's effect on its deficits. When assessing these deficiencies, it is also important to evaluate all possible causes, including efferent nerve signals. 

*Electromyography* 

Electromyography (EMG) may serve as a supportive tool for assessing neuromuscular deficiency considering the major nerve fibers that cross graft sites, but it is not commonly assessed. An initial investigation in 2019 by Letter et al. identified no major latency deficit between the ipsilateral and contralateral quadriceps [43]. Subsequently, a revisit in 2022 by Ito et al. [44] calculated a meaningful difference in vastus medialis latency of the affected quadriceps at baseline and two years post-surgery. Although a less commonly measured aspect of graft-site morbidity, EMG shows some promise as a future tool. Dynamometers are a robustly used instrument in the measurement of muscle deficits due to graft-site morbidity. 

*Dynamometer* 

Dynamometers are a quantitative tool that some researchers used to assess muscle strength recovery after graft harvest. There were 17 studies that used dynamometry to assess postoperative strength (Figure 2F). Testing protocols differed in angular velocity settings and repetition schemes. Two manuscripts measured patient performance exclusively at the 60 deg/sec [8,21], while one manuscript measured patient performance at 90 deg/sec interval [23]. Fourteen of the manuscripts included multiple settings from a range of 60 deg/sec to 300 deg/sec, but their selected observation intervals rarely matched [10,12,21,25-27,29,30,40,42,45-48]. Studies selected 60 deg/sec as the most common observation interval. Researchers continued to divide themselves based on repetitions of testing ranging from three repetitions to 30 [20,29] In three studies, patients warmed up on a bike or ergometer before beginning their testing series [21, 29,44]. Consistency in the speed intervals is severely lacking in the current studies, which precludes further statistical analysis.

Discussion

According to current research, each graft type has variable advantages in surgical outcomes, with each providing adequate tensile strength to support knee movement in lieu of the ACL [49]. While this may be an acceptable minimum outcome, it is important to include patient well-being in our overall selection of graft site. Individual researchers have identified differences based on graft-site morbidity, especially when evaluating anterior knee pain, paresthesia, and muscle strength. However, the inconsistencies in methodology stall any effort to meaningfully link these data sets together through meta-analysis.

Anterior knee pain can significantly detract from quality of life, including loss of athletic performance, limiting job opportunities, and difficulties performing daily tasks. However, the subjective nature of pain makes it inherently challenging to measure. Researchers have approached this problem from a multitude of angles, ranging from a binary presentation to comprehensive questionnaires that provide a composite score of pain symptoms. Scoring systems that include questions on functional limitations such as pain with running, jumping, walking, or rest can provide better insight than a subjective numbering system. This functional approach provides benchmarks of motor activity that the physician can use to track the progression and elimination of pain. Ultimately, the goal of ACLR is a return to full knee functionality, and pain should be readily considered in the evaluation of a successful surgery. 

The kneeling test is another tool to measure the functional impact of anterior knee pain. Without the ability to kneel, even the simple task of tying one's shoe becomes a significant obstacle in day-to-day life. Moreover, patients could struggle with jobs requiring kneeling, which can jeopardize financial security and safety nets such as insurance coverage. Patients may also face difficulties when observing religious practices if they have pain with kneeling. These hinderances can reduce life satisfaction and challenge a patient's sense of identity. Graft site selection may play a role in reducing these kneeling symptoms, suggesting the need for cohesive investigation strategies. 

Another major concern during graft harvesting should be paresthesia since there are a multitude of nerve structures passing near the excision site. There is limited literature identifying significant nerve structures within the incision path to access the QT graft [50]. In contrast, the site of the BPTB graft crosses the infrapatellar branch of the saphenous nerve, which provides the afferent sensation of the anterior knee [50-52]. This poses a significant risk of paresthesia during graft harvesting. Physicians can quantitatively assess the area of paresthesia by encouraging the patient to outline the area of altered sensation with a marker. Consistent use of these methods would provide the opportunity to objectively compare areas of nerve damage from graft harvesting.

While pain and paresthesia critically contribute to the patient's experience, functional tests like the hop test, EMG, and dynamometer can eliminate some of the subjectivity inherent to the previous measures. The linear distance hop test assesses each leg individually, and it requires both flexor and extensor actions to complete the exercise. While this complex activity is likely only achievable in the later months of recovery, it is a quantifiable test to measure regaining extensor muscle strength. This test does not require expensive instruments to complete, serving as a good measurement for any clinic regardless of funding. Future studies should clearly differentiate between this test and the knee stability hop test, which assesses ACL integrity rather than the graft site. The functional hop test is an excellent test for researchers looking for a budget-friendly assessment tool.

EMG is an area that has not been thoroughly explored, but it has good potential for assessment of graft-site morbidity. EMG is low risk and can be used in the earliest stages of recovery to assess a patient's retention of efferent nerve signal to the graft site [44]. It could complement the other functional tests well to determine if a patient's graft-site morbidity is due to nerve transection. Few papers have explored the use of EMG testing in measuring graft-site morbidity, but this may be an area of interest for future quantitative assessment.

The dynamometer may serve as a good candidate for quantitative and consistent measurement of regaining muscle strength. It provides real-time objective feedback for the observer to assess graft site recovery through the rehabilitation process. The adjustability of the dynamometer allows the physician to measure torque output at various levels of endurance. This allows alignment of the rehabilitation process with patient goals regarding power and endurance. However, this flexibility has also created substantial variation in the current literature, limiting comparisons across studies. To generate a meta-analysis, a standardized approach should be implemented in dynamometer usage that assesses both power and endurance. 

While evaluation of all graft-site morbidity outcomes currently evades meta-analysis, other statistical approaches may be suitable to increase the sum of their impact. Graft-site morbidity factors could be split into smaller subgroups according to their assessment technique to aid the reviewer in compilation of similarly measured data points. While the power of these data sets would be reduced, the resulting meta-analysis may better delineate more specific or sensitive assessment techniques. Another statistical strategy would require the reviewer to normalize the data by transforming to a common effect; i.e. standard deviation or multiple of the median. This strategy could be limited by incorrect transformation, persistent heterogeneity of outcome, and distortion of result outcomes. In the absence of standardized techniques, future reviewers rely on creative and methodologically limited approaches to synthesizing evidence, underscoring the need for consensus of graft-site morbidity assessment. 

## Conclusions

Overall, significant references to graft-site morbidity outcomes were identified in the initial literature search, indicating their importance in patient recovery from autograft ACLR. However, the current variance in methodology limits the potential of combining these data sets for meta-analysis. The reviewers propose the need for standardization of the current measurement techniques to evaluate anterior knee pain, paresthesia, and muscle strength to generate standardized data surrounding graft-site morbidity. The ability to perform a meta-analysis in the future will provide invaluable information on graft-site morbidity outcomes, allowing surgeons and patients to choose a graft that best suits each patient's goals and lifestyle.
